# The implementation of the mobile application “Colorectal Leakage App”, based on the Dutch Leakage Score, for early detection of anastomotic leakage after colorectal surgeries—pilot study

**DOI:** 10.3389/fsurg.2025.1538023

**Published:** 2025-06-04

**Authors:** Meiram Mamlin, Saule Khamzina, Gulden Zhanmukanbetova, Nurlan Mukazhanov, Aidana Amangeldiyeva, Arman Kozhakhmetov

**Affiliations:** ^1^ Department of Multidisciplinary Surgery, National Research Oncology Center, Astana, Kazakhstan; ^2^ Mathematics in Data Science, Technical University of Munich, Munich, Bavaria, Germany; ^3^ Resident of Chemotherapy Department, National Research Oncology Center, Astana, Kazakhstan; ^4^ Department of Medical Affairs, Pfizer Export B. V., Almaty, Kazakhstan; ^5^ School of Medicine, Nazarbayev University, Astana, Kazakhstan

**Keywords:** anastomotic leak (AL), colorectal cancer, postoperative monitoring, early detection, mobile application

## Abstract

**Background:**

Colorectal cancer is the third most common malignancy globally and in Kazakhstan, with anastomotic leakage (AL) being a severe complication of colorectal surgeries. Early detection of AL is critical for improving patient outcomes and reducing mortality, as the condition is also characterized by a deterioration in quality of life. The “Colorectal Leakage App,” based on the Dutch Leakage Score (DLS), offers a standardized approach for postoperative monitoring and early AL detection.

**Methods:**

In this single-center pilot study conducted at the National Research Oncology Center in Kazakhstan, sixty-three patients who underwent colorectal surgery were monitored daily during the postoperative period using the “Colorectal Leakage App” mobile application. Patients with anastomosis were included. The app integrates clinical signs and laboratory data to recommend further diagnostic steps, such as CT with rectal contrast. The primary endpoint was to determine the feasibility of using the “Colorectal Leakage App” in standardized postoperative care. The frequency of AL was also measured as an additional outcome.

**Results:**

Of 63 patients enrolled, AL was observed in 3.2% (2/63) of cases. In one case, the app flagged a score of 9 on postoperative day (POD) 7, prompting timely CT and intervention. Another case was identified via drainage findings on POD 5. A third flagged case (score 10, POD 10) revealed a gallbladder abscess rather than AL, highlighting the app’s potential for broader complication monitoring.

**Discussion:**

The “Colorectal Leakage App,” based on the Dutch Leakage Score, was integrated as a standardized postoperative care protocol. Given the small sample size and insufficient number of AL cases, statistical analysis is currently not feasible. However, initial results suggest that the application may have a role in enhancing postoperative surveillance.

**Conclusion:**

The implementation of the “Colorectal Leakage App” may facilitate the early detection of AL. In this single-center pilot study, the AL rate was 3.2% (2 out of 63 patients). We plan to continue our study and conduct a multicenter study to further evaluate the app's effectiveness across different healthcare settings in Kazakhstan, aiming to standardize postoperative care.

## 1 Introduction

Colorectal cancer ranks third among oncological diseases worldwide in both men and women (1), and it holds the same position in Kazakhstan (2). Anastomotic leakage (AL) following colorectal surgeries is one of the most serious complications arising from colorectal surgical interventions. This condition is characterized by increased mortality and significant deterioration in the quality of life of patients (3). Additionally, the presence of AL after anterior resections for rectal cancer is associated with an increased risk of local disease recurrence (4). The frequency of AL varies between 2% and 19%, depending on risk factors, AL definition, and the level of anastomosis (5–8). Given the high likelihood and severe consequences of AL, a relevant task is its early detection and effective treatment.

Clinical signs of AL traditionally include fever, tachycardia, pain syndrome, purulent or fecal discharge through drainage, and dynamic bowel obstruction (9). These signs, combined with other clinical data, are integrated into a diagnostic-specific indicator known as the Dutch Leakage Score (DLS) (10). This indicator is used as a standardized postoperative monitoring protocol for patients suspected of having AL, aiming to minimize delays in detecting this complication and, consequently, reduce early postoperative mortality.

In this pilot study, we aim to assess the feasibility of the mobile application “Colorectal Leakage App” in the early detection of AL after colorectal surgeries. The study has been being conducted at the National Research Oncology Center (NROC) in Astana, Kazakhstan, since January 2024. The primary goal is to assess the feasibility of using the “Colorectal Leakage App” for detecting AL after colorectal surgeries.

It is important to note that there is currently no up-to-date data on the number of colorectal cancer surgeries performed in Kazakhstan, and information on the frequency of anastomotic leakage (AL) after colorectal surgeries in this population is also lacking. Previous studies have shown that the effectiveness of the DLS algorithm is directly proportional to the frequency of AL (10). Therefore, it is assumed that the application of DLS may demonstrate different results in the conditions of Kazakhstan, which we also plan to measure through the “Colorectal Leakage App” mobile application.

## 2 Materials and methods

### 2.1 Study design

In this single-center pilot study, sixty-three patients who underwent colorectal surgery were monitored daily during the postoperative period using the “Colorectal Leakage App” mobile application. Patient recruitment began in January 2024. Patients were included in the study after providing informed consent. The study protocol was approved by the hospital's Ethical and Scientific Review Board (Approval No. 25) and registered in an open-access database (https://www.clinicaltrials.gov: NCT06273826).

### 2.2 Criteria for selecting research participants

The study includes both male and female individuals aged 18 years and older. Participants from various national and ethnic backgrounds are considered.

The inclusion criteria specify that participants must be scheduled for surgical treatment of benign or malignant colorectal diseases. This includes procedures involving the creation of ileocolic, colocolic, or colorectal anastomoses, with or without a protective stoma. Eligible participants must have an Eastern Cooperative Oncology Group (ECOG) performance status of 0–2, along with satisfactory hematological indicators and adequate liver and kidney function.

Exclusion criteria are as follows: individuals under the age of 18, pregnant or lactating participants, those planning pregnancy during the study period, and those with an ECOG performance status above 2 or with unresectable tumors. Furthermore, any participant who chooses to withdraw from the study will also be excluded.

This study does not include vulnerable populations, ensuring ethical standards are maintained in the selection of research participants.

### 2.3 The “Colorectal Leakage App”

**Figure 1:**
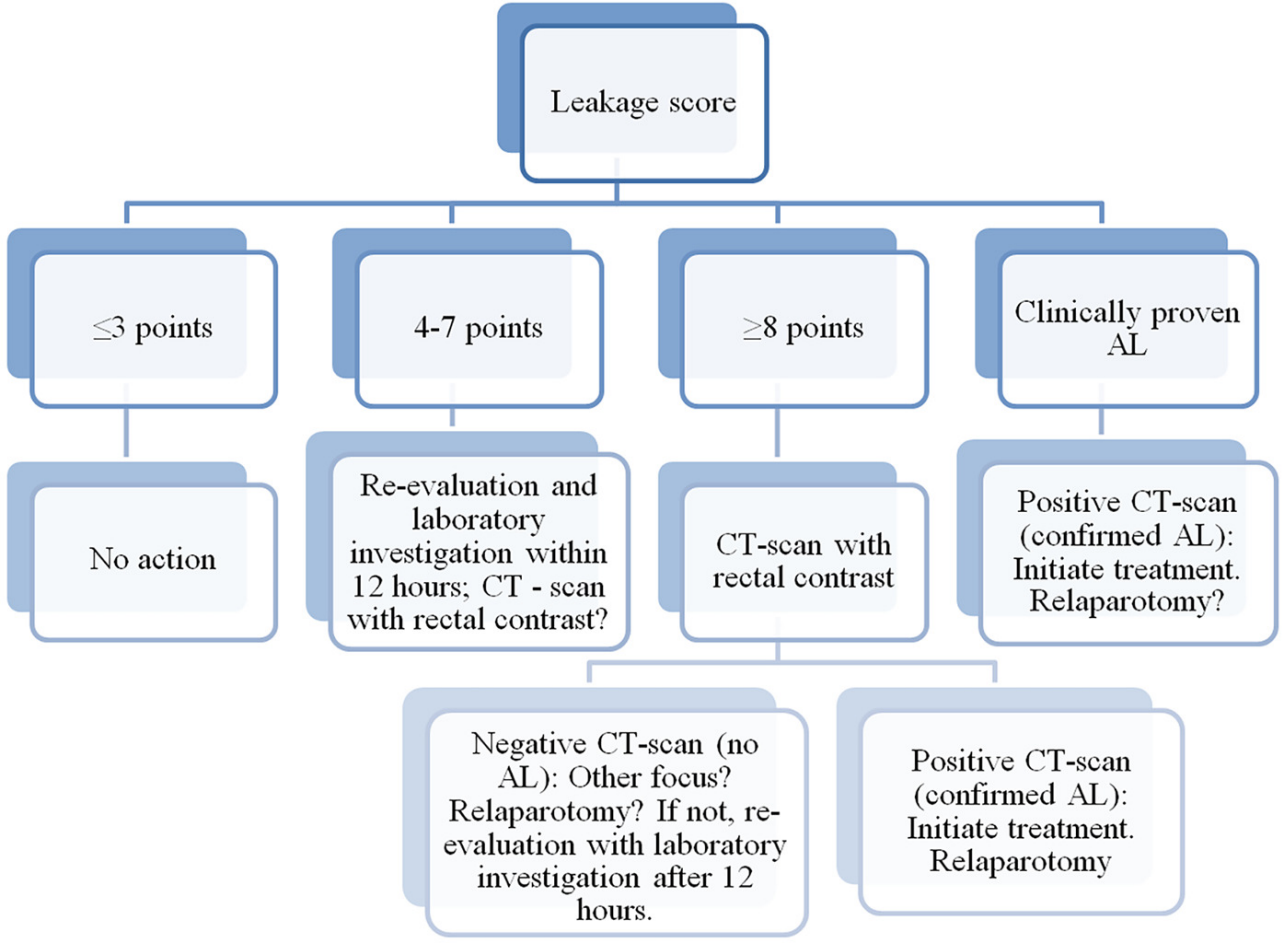
Algorithm for assessing anastomotic leakage, indicating actions to be taken for each result. Clinically proven anastomotic leakage (fecal discharge in drainage or wound) is treated identically to confirm anastomotic leakage on CT scan. AL, anastomotic leakage. CT, computed tomography.

The “Colorectal Leakage App” is a mobile application that, based on an algorithm, provides an assessment of the risk of AL in patients after colorectal surgeries. The algorithm is built on the standardized postoperative monitoring protocol called “Dutch Leakage Score” (Table 1). After calculating the scores, the application provides recommendations for further actions (Figure 1).

**Table 1:** Items of Dutch leakage score.

Item	Normal value	Score	Abnormal value	Score
General				
Fever	≤38.0°C	0	>38.0°C	1
Heart rate	≤100/min.	0	>100/min.	1
Respiratory rate	≤30/min.	0	>30/min.	1
Urinary production	≥30 ml/hour or 700 ml/day	0	<30 ml/hour or 700 ml/day	1
Mental status	Normal	0	Agitation or lethargic	2
Clinical condition	Stable or improving condition	0	Deterioration	2
Local physical examination				
Signs of ileus	No ileus	0	Ileus	2
Gastric retention	No gastric retention	0	Gastric retention	2
Fascial dehiscence	No fascial dehiscence	0	Fascial dehiscence	2
Abdominal pain, other than wound pain	No pain other than wound pain	0	Pain other than wound pain	2
Laboratory investigation				
Signs of infection	No increase in leukocyte number or CRP	0	Increase in leukocyte number or CRP ≥ 5%.	1
Kidney function	No increase in urea or creatinine	0	Increase in urea or creatinine ≥5%.	1
Diet				
Nutritional status	Normal diet	0	Tube feeding/TPN	1/2

The application includes an algorithm based on the DLS. Each postoperative day, data entry at the patient's bedside is carried out in the application. Based on the data analysis, the algorithm provides recommendations regarding the necessity of conducting a computer tomography (CT) with rectal contrast (Figure 1).

The following information is also collected: Preoperative risk factors for AL (age, gender, obesity, nutritional status, diabetes, cardiovascular diseases, renal insufficiency, inflammatory bowel diseases, ASA score, bowel preparation); Surgery data (access, surgery type, anastomosis technique, duration of surgery, blood loss volume, creation of a preventive stoma, intraoperative and postoperative complications, pTNM).

Key points determining the conclusion of a patient's participation in the study are: the detection of AL, patient discharge from the hospital, or patient death.

### 2.4 Study endpoints

The primary endpoint of this pilot study is to determine the feasibility of using the “Colorectal Leakage App” in standardized postoperative care. Additionally, the frequency of AL following colorectal surgeries is assessed.

Secondary endpoints focus on related outcomes, including mortality within 30 days post-surgery and the delay in diagnosing AL, measured as the number of days from the onset of first signs of leakage to the confirmed diagnosis of AL. The overall duration of hospital stay, the number of days spent in the intensive care unit (ICU), and the interval in days between the surgery and the detection of AL are also tracked in the study.

### 2.5 Statistical analyses

Data were analyzed with the SPSS package (SPSS 23.0 for Windows; SPSS Inc, Chicago, IL). Quantitative values are expressed as median values and Interquartile Range (IQR). Categorical data are shown with percentage frequencies.

## 3 Results

At this point 63 patients have been recruited with a median age of 64 years, (IQR 13) (Table 2). The cohort had a slight predominance of females (35 patients, 55.6%) compared to males (28 patients, 44.4%). The majority of patients (87.3%, 55/63) underwent surgery for cancer, with 3.2% (2/63) for benign disease and 9.5% (6/63) for stoma reversal. All patients underwent colon preparation with PEG and metronidazole per os, and the surgical approach was divided between open surgery (31/63, 49.2%), laparoscopic surgery (29/63, 46%), and video-assisted surgery (3/63, 4.8%). Conversion from laparoscopic to open surgery occurred in 7 patients (24.1%). Various surgical procedures were performed, the most common being low anterior resection (14/63, 22.2%), sigmoidectomy (13/63, 20.6%), right hemicolectomy (8/63, 12.7%), anterior resection (8/63, 12.7%), left hemicolectomy (6/63, 9.5%), end colostomy reversal (4/63, 6.3%) and other less frequent resections. The median duration of hospital stay for participants was 8 days, with IQR of 2 days. The number of days spent in ICU had a median of 1 day and an IQR of 0.0 day. Table 2 outlines all demographic patient characteristics.

**Table 2:** Patient characteristics.

Parameter	Category	Value (*n* = 63)
		Median (IQR) or *n* (%)
Age, years		64 (13)
Sex	Female	35 (55.6%)
	Male	28 (44.4%)
BMI	<25	22 (34.9%)
	≥25, 30	27 (42.9%)
	≥30, <35	11 (17.5%)
	≥35, <40	2 (3.1%)
	≥40	1 (1.6%)
Diabetes Type 2	No	53 (84.1%)
	Yes	10 (15.9%)
Cardiovascular disease	No	23 (36.5%)
	Yes	40 (63.5%)
Renal failure	No	60 (95.2%)
	Yes	3 (4.8%)
ASA	ASA 1	13 (20.6%)
	ASA II	50 (79.4%)
Indication for surgery	Benign disease	2 (3.2%)
	Cancer	55 (87.3%)
	Stoma reversal	6 (9.5%)
Localization	Appendix	1 (1.6%)
	Caecum	3 (4.8%)
	Ascending colon	2 (3.2%)
	Liver flexure	1 (1.6%)
	Splenic flexure	7 (1.1%)
	Descending colon	4 (6.3%)
	Sigmoid colon	17 (27%)
	Rectosigmoid colon	8 (12.7%)
	Rectum	17 (27%)
Colon preparation	PEG + metronidazole per os	63 (100%)
Surgical approach	Open	31 (49.2%)
	Laparoscopic	29 (46%)
	Video assisted	3 (4.8%)
Conversion	No	22 (75.9%)
	Yes	7 (24.1%)
Surgery	Low anterior resection	14 (22.2%)
	Sigmoidectomy	13 (20.6%)
	Anterior resection	8 (12.7%)
	Right hemicolectomy	8 (12.7%)
	Left hemicolectomy	6 (9.5%)
	End colostomy reversal	4 (6.3%)
	Splenic flexure resection	2 (3.2%)
	Extended left hemicolectomy	1 (1.6%)
	Left hemicolectomy with anterior resection	1 (1.6%)
	End ileostomy reversal	1 (1.6%)
	Intersphincteric resection	1 (1.6%)
	Subtotal colectomy	1 (1.6%)
	Preventive ileostomy reversal with ileocolon anastomosis	1 (1.6%)
	Subtotal colectomy with low anterior resection	1 (1.6%)
Duration of hospital stay		8 (2)
Days spent in ICU		1 (0)
Anastomosis technique	Hand-sewn	24 (38.1%)
	Stapled	39 (61.9%)
Anastomosis type	End-to-end	26 (41.3%)
	End-to-side	4 (6.3%)
	Side-to-side	13 (20.6%)
	No information	1 (1.6%)
Duration	<240 min	50 (79.4%)
	≥240 min	13 (20.6%)
Blood loss	<200 ml	55 (87.3%)
	≥200 ml	8 (12.7%)
Loop ileostomy	No	46 (73%)
	Yes	17 (27%)
Simultaneous surgery	No	54 (85.7%)
	Yes	9 (14.3%)
Complications	Overall	12 (19%)
Clavien-Dindo grade	CD 1	3 (4.7%)
	CD 2	6 (9.5%)
	CD 3	3 (4.7%)
Anastomotic leakage	No	61 (96.8%)
	Yes	2 (3.2%)

Anastomosis was performed using hand-sewn techniques in 38.1% (24/63) of cases, while stapled anastomosis was used in 61.9% (39/63). The most common anastomosis type was end-to-end (41.3%, 26/63), followed by side-to-side (20.6%, 13/63) and end-to-side (6.3%, 4/63). Most surgeries (79.4%, 50/63) had a duration of less than 240 min, while 13 cases (20.6%) took longer than 240 min. Blood loss was minimal in most surgeries, with 87.3% (55/63) losing less than 200 ml of blood. A protective loop ileostomy was created in 27% (17/63) of cases, and simultaneous procedures were performed in 14.3% (9/63). Postoperative complications were documented in 19% (12/63) of patients, classified by the Clavien-Dindo (CD) as Grade 1 in 4.7% (3/63), Grade 2 in 9.5% (6/63), and Grade 3 in 4.7% (3/63), with no Grade 4 or 5 complications. Anastomotic leakage (AL) was observed in 3.2% (2/63) of cases.

**Figure 2:**
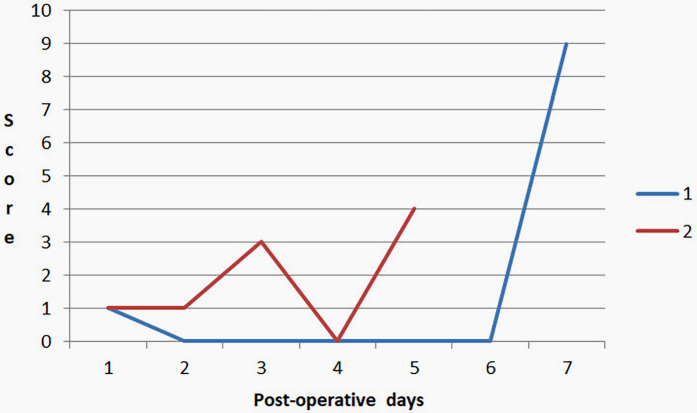
Trends in DLS scores recorded by the app during the postoperative period in both patients with AL.

At the moment, two cases of AL have been documented. In the first case, leakage was identified through the mobile application “Colorectal Leakage App” on postoperative day (POD) 7, the application generated a score of 9, prompting a pelvic CT scan with rectal contrast. The imaging confirmed the leakage, and the patient was taken for surgical intervention. Therefore, there was no delay in the diagnosis of AL. In the second case, the application demonstrated a score of 4 and fecal discharge was observed via a pelvic drain on POD 5, which was interpreted as an AL, leading to surgical management. Figure 2 shows the trends in DLS scores recorded by the app during the postoperative period in both patients. Both had diabetes mellitus, cardiovascular diseases, and obesity. They each underwent elective open sigmoidectomy—one with a hand-sewn anastomosis and the other with a stapled anastomosis. Additionally, there was one case where a score of 10 was recorded on POD 10. A CT scan with contrast was performed, revealing an abscess in the gallbladder bed without evidence of AL.

## 4 Discussion

Today, the mobile phone has become an extension of the human hand. With the widespread use of smartphones and applications in almost every field, it seems logical to extend this technology into the realm of surgery. Mobile apps have the potential to greatly enhance surgeons' efficiency by simplifying routine patient monitoring and offering real-time, algorithm-based recommendations. These intelligent systems could assist in decision-making, ensuring timely interventions and optimizing patient care, while reducing the cognitive load on surgeons. This would be particularly beneficial in low-volume hospitals. By implementation of mobile technology, these clinics could improve early diagnosis, which would in turn help reduce both mortality and morbidity. Furthermore, this approach would be highly cost-effective by reducing the severity of postoperative complications and shortening patients' hospital stays, ultimately improving both clinical outcomes and healthcare resource utilization.

Using mobile apps in surgery could also standardize postoperative care, making it easier for surgeons to track a patient's progress through digital checklists, and data collection. Ultimately, this technology would enhance patient outcomes, allow more efficient use of time, and reduce the likelihood of complications due to delayed diagnoses. F Jasmijn Smits, Anne Claire Henry et al. implemented an algorithm based mobile application for early detection of complications after pancreatic resection what considerably improved clinical outcomes compared with usual care This difference included an approximate 50% reduction in mortality at 90 days (11).

We integrated DLS, developed by M. den Dulk et al., into our mobile application, the “Colorectal Leakage App”. In their study, M. den Dulk and colleagues demonstrated a reduction in the delay of AL detection from 4 days to 1.5 days. The original score consisted of 11 items, but it was later refined to 4 items, focusing on three clinical parameters and a C-reactive protein (CRP) level above 250 mg/L (12). The initial version of the score was incorporated into our application to gather more data for future analysis.

The ICRAL study further validated the DLS as an excellent predictor of AL, with the Area Under the Receiver Operating Characteristic (ROC) Curve for predicting AL on postoperative days (POD) 2 and 3 being 0.75 and 0.84, respectively (13).

In our study the identification of AL in two cases highlights both the critical nature of early detection and the potential value of “Colorectal Leakage App.” In the first case, the app played a role in identifying AL on the 7th postoperative day, prompting a timely pelvic CT with rectal contrast, which confirmed the leakage and enabled immediate surgical intervention. The use of mobile applications for clinical monitoring, as demonstrated here, can enhance early detection of complications that might otherwise be missed or delayed.

In the second case the application's score 4 and fecal discharge through a pelvic drain, led to the diagnosis of AL on the 5th postoperative day.

Additionally, in the third instance, a score of 10 was recorded on postoperative day (POD) 10 via the app, but subsequent CT imaging revealed an abscess in the gallbladder bed rather than an AL. This case highlights the challenges of false positives and the potential for non-specific findings in postoperative patients when using the app. Nonetheless, it also demonstrates how standardized postoperative monitoring through the app can facilitate the early identification of other complications, prompting timely interventions. In other words, the mobile application may serve as a daily screening for sepsis in patients after colorectal surgeries as SIRS criteria are included in the algorithm.

During the implementation of the application in our daily practice, we found it convenient for monitoring patients in the postoperative period due to its standardized format. Moreover, it proved to be an effective teaching tool for students and residents during their clinical rotations, helping us to unify and streamline postoperative care. It is particularly useful in complex cases, where multiple aspects of the patient's condition require consistent and thorough evaluation.

Given the small sample size and insufficient number of AL cases, statistical analysis is currently not feasible, limiting the ability to draw generalized conclusions from this data. However, these cases suggest that the application may have a role in enhancing postoperative surveillance.

Another limitation of this study is the heterogeneity of the patient group, including those with and without a protective stoma. However, the creation of a protective stoma after low anterior resections is now a routine component of such procedures (14). For this reason, we considered it essential to include these patients in the study, as anastomotic leakage can still occur in this group. Moreover, even in the presence of a stoma, clinically significant leaks may still necessitate additional interventions or reoperations.

Our future plans include to continue our research and to conduct a multicenter study aimed to investigate AL frequency in other hospitals of Kazakhstan with further implementation of “Colorectal leakage app” in their standard postoperative care. Additionally, we plan to leverage deep learning (DL) to analyze data from the app and optimize the items of the DLS score. The integration of DL, offers transformative potential in medicine and biomedical research, including colorectal cancer (15).

## 5 Conclusion

The implementation of the “Colorectal Leakage App” may facilitate early detection of AL. In this single-center pilot study, the AL rate was 3.2% (2 out of 63 patients). While the sample size limits any statistical conclusions, these initial findings suggest that integrating mobile technology with standardized monitoring protocols like the Dutch Leakage Score may improve patient outcomes. We plan to continue our study and conduct multicenter study to further evaluate the app's effectiveness across different healthcare settings in Kazakhstan, aiming to standardize postoperative care.

## Data Availability

The raw data supporting the conclusions of this article will be made available by the authors, without undue reservation.
